# Genomic epidemiology of *Escherichia coli*: antimicrobial resistance through a One Health lens in sympatric humans, livestock and peri-domestic wildlife in Nairobi, Kenya

**DOI:** 10.1186/s12916-022-02677-7

**Published:** 2022-12-08

**Authors:** Dishon M. Muloi, James M. Hassell, Bryan A. Wee, Melissa J. Ward, Judy M. Bettridge, Velma Kivali, Alice Kiyong’a, Christine Ndinda, Nduhiu Gitahi, Tom Ouko, Titus Imboma, James Akoko, Maurice K. Murungi, Samuel M. Njoroge, Patrick Muinde, Lorren Alumasa, Titus Kaitho, Fredrick Amanya, Allan Ogendo, Bram A. D. van Bunnik, John Kiiru, Timothy P. Robinson, Erastus K. Kang’ethe, Samuel Kariuki, Amy B. Pedersen, Eric M. Fèvre, Mark E. J. Woolhouse

**Affiliations:** 1 Usher Institute, University of Edinburgh, Edinburgh, UK; 2 Centre for Immunity, Infection and Evolution, University of Edinburgh, Edinburgh, UK; 3 International Livestock Research Institute, Nairobi, Kenya; 4 Institute of Infection, Veterinary and Ecological Sciences, University of Liverpool, Neston, UK; 5 Nuffield Department of Clinical Medicine, University of Oxford, John Radcliffe Hospital, Oxford, UK; 6 Faculty of Medicine, University of Southampton, Southampton, UK; 7 Natural Resources Institute, University of Greenwich, Chatham Maritime, UK; 8 University of Nairobi, Nairobi, Kenya; 9 Centre for Microbiology Research, Kenya Medical Research Institute, Nairobi, Kenya; 10 National Museums of Kenya, Nairobi, Kenya; 11 Veterinary Services Department, Kenya Wildlife Service, Nairobi, Kenya; 12 Animal Production and Health Division, Food and Agriculture Organization of the United Nations, Rome, Italy; 13 Institute of Evolutionary Biology, School of Biological Sciences, University of Edinburgh, Edinburgh, UK

**Keywords:** Antimicrobial resistance, AMR, One Health, *Escherichia coli*, Genomics

## Abstract

**Background:**

Livestock systems have been proposed as a reservoir for antimicrobial-resistant (AMR) bacteria and AMR genetic determinants that may infect or colonise humans, yet quantitative evidence regarding their epidemiological role remains lacking. Here, we used a combination of genomics, epidemiology and ecology to investigate patterns of AMR gene carriage in *Escherichia coli*, regarded as a sentinel organism.

**Methods:**

We conducted a structured epidemiological survey of 99 households across Nairobi, Kenya, and whole genome sequenced *E. coli* isolates from 311 human, 606 livestock and 399 wildlife faecal samples. We used statistical models to investigate the prevalence of AMR carriage and characterise AMR gene diversity and structure of AMR genes in different host populations across the city. We also investigated household-level risk factors for the exchange of AMR genes between sympatric humans and livestock.

**Results:**

We detected 56 unique acquired genes along with 13 point mutations present in variable proportions in human and animal isolates, known to confer resistance to nine antibiotic classes. We find that AMR gene community composition is not associated with host species, but AMR genes were frequently co-located, potentially enabling the acquisition and dispersal of multi-drug resistance in a single step. We find that whilst keeping livestock had no influence on human AMR gene carriage, the potential for AMR transmission across human-livestock interfaces is greatest when manure is poorly disposed of and in larger households.

**Conclusions:**

Findings of widespread carriage of AMR bacteria in human and animal populations, including in long-distance wildlife species, in community settings highlight the value of evidence-based surveillance to address antimicrobial resistance on a global scale. Our genomic analysis provided an in-depth understanding of AMR determinants at the interfaces of One Health sectors that will inform AMR prevention and control.

**Supplementary Information:**

The online version contains supplementary material available at 10.1186/s12916-022-02677-7.

## Background

Rising levels of bacterial infections resistant to last-line antimicrobial agents represent a global health crisis [1], and the role of livestock in rising levels of antimicrobial-resistant bacterial infections observed in humans has been the subject of much speculation [2]. Humans and livestock are linked in many ways, including direct contact through agriculture, consumption of livestock products by humans and shared environments contaminated with human sewage and manure from livestock.

The links between human and livestock populations provide an opportunity for either population to act as a reservoir from which antimicrobial-resistant bacteria or their antimicrobial resistance (AMR) determinants could be transmitted in either direction [3]. Wildlife has also been documented to carry antimicrobial-resistant bacteria [4] and can contact both humans and livestock in a range of different environments [5]. The role of livestock keeping in the emergence and transmission of AMR bacteria to human (and potentially wildlife) populations is still not well understood.

Cities in low-income countries have been posited as ‘melting-pots’ of both infectious disease and AMR, due to poor hygiene and sanitation, densely populated human settlements found alongside livestock and a rich assortment of wildlife, and largely unregulated antimicrobial usage. Here, we focused on the role of livestock keeping within households across Nairobi, Kenya, as a high-risk urban interface for the emergence and transmission of AMR bacteria between humans and animals. We use *Escherichia coli*, a common commensal and pathogenic bacterium [6] in vertebrates, to investigate the dispersal of AMR between human and animal hosts in 99 households across Nairobi using an epidemiologically structured analytical framework.

Current surveillance of AMR tends to focus on tracking specific resistance phenotypes and genotypes separately in human and animal populations, without making epidemiological comparison of resistances between the two [7]. The application of high-resolution whole genome sequencing on spatiotemporally related isolates may help us to improve our understanding of drivers of AMR dispersal across the human-animal interface [8]. Here, using whole genome sequence analysis of *E. coli* isolates obtained from cohabiting human and animal populations, we determined the prevalence and mechanisms of resistance and characterised AMR gene diversity and structure of AMR genes in different host populations across the city. At a finer scale of individual households, we use ecological models to investigate risk factors for the exchange of AMR genes between sympatric humans and livestock, thus shedding light on pathways of AMR transfer at household interfaces.

## Methods

### Study design

A cross-sectional study targeting synanthropic wildlife and sympatric human and livestock populations in Nairobi, Kenya, was carried out from August 2015 to October 2016 as part of the Urban Zoo Project [9]. Briefly, Nairobi city was stratified into administrative sublocations according to socioeconomic status, identifying 70 possible sub-locations. Thirty-three sub-locations were chosen to maximise spatial distribution, socio-economic diversity and attempt to capture the diversity of livestock-keeping practices across the city [10]. For each sub-location, three households—two livestock keeping (small livestock only (poultry, rabbits and goats) and large livestock (cattle and pigs) with or without small livestock) and one non-livestock-keeping—were selected at random within the dominant housing type. A total of 99 households were involved in the study (Additional file 1: Figure S1).

### Sample collection and microbiological methods

In each household, a questionnaire was used to collect data on (i) household composition, food consumption, medical history and socio-economic variables and (ii) livestock ownership and management practices. The household area including outdoor spaces (metres^2^) was measured using ArcGIS and livestock and human abundance data derived from each household. Details of taxon-specific methods used to trap wildlife and to collect samples from humans and animals are described in the supplementary methods and elsewhere [9]. Collected faecal samples were transported on ice to one of two laboratories (University of Nairobi or Kenya Medical Research Institute) within 5 h of collection. Samples were enriched in buffered peptone water for 18 h and plated onto eosin methylene blue agar and incubated for 18 h at 37 °C. One colony from each plate was selected and sub-cultured for a further 18 h on the second round of EMBA. Subsequently, one purified colony from each plate was selected at random (hereafter referred to as an ‘isolate’) and confirmed as *E. coli* by biochemical testing, using triple sugar iron agar, Simmons citrate agar and motility-indole-lysine media.

### Whole-genome sequencing

DNA was extracted from bacterial isolates using commercial kits (Purelink® Genomic DNA Mini Kit, Invitrogen, Life Technologies, Carlsbad, CA) at the International Livestock Research Institute, Nairobi, Kenya, and transported under licence to The Wellcome Trust Centre for Human Genetics, Oxford, UK. Whole-genome sequencing was carried out at the Wellcome Trust Centre for Human Genetics on the Illumina HiSeq 2500 platform.

### Bioinformatic analysis

Sequenced reads were filtered for quality and trimmed for adaptors with BBDuk (v38.46), k = 19 mink = 11 hdist = 1 ktrim = r minoverlap = 12 qtrim = rl trimq = 15. The following sequencing quality thresholds were used based on Quast: (i) at least 3 Mb aligned to EC958, (ii) a maximum assembly length of 6.5 Mb, (iii) GC content of between 50 and 51% and (iv) assembly N50 of > 30 kb or a maximum of 100 cgMLST missing loci. A total of 1316 genomes passed this quality threshold.

### Detection of antimicrobial resistance genes

Acquired antibiotic resistance genes were identified from the assemblies using starAMR [11], with percent identity (> 95%) and coverage (> 60%), against the ResFinder database downloaded on 25 September 2019 [12]. Chromosomal mechanisms of fluoroquinolone resistance were identified by screening isolates using PointFinder [13] for the presence of associated amino acid changes in the quinolone resistance-determining regions of *gyrA* and *parC* alleles.

### Distribution of AMR genes by host types

Hosts were categorised into five broad types: (i) humans; (ii) livestock birds, poultry dominated by chickens; (iii) livestock mammals consisting of ruminants and monogastric livestock; (iv) wild birds, predominantly seed-eating birds such as house sparrows; and (v) Wild mammals, predominantly rodents and bats. Differences in the distribution of AMR genes between hosts were calculated using the chi-squared tests and one-way ANOVA using R package stats. Tukey’s multiple-comparison test was performed post hoc for pairwise comparisons between the groups, and *p* values of < 0.05 were considered significant.

### Alpha diversity

Comparisons of alpha diversity between host groups were conducted using the Richness index (observed richness) (defined as the number of unique AMR genes in an isolate), Simpson diversity index and Shannon index using the diversity function in the vegan package [14]. The Kruskal–Wallis test was applied, and statistical differences were corrected for multiple comparisons using a Bonferroni correction.

### Rarefaction analysis

To estimate whether or not differential sampling bias could be, in part, responsible for the observed diversity of resistance determinants in the isolates, we performed a sample-based rarefaction analysis and rank abundance analysis [15] using the package iNEXT [16]. Rarefaction extrapolation curves were plotted using a doubling in sample size as defined by Chao and Jost [17], and 999 bootstrap replicates were used to estimate 95% confidence intervals.

### Co-occurrence network of acquired resistance genes

Co-occurrence patterns among pairs of acquired AMR genes based on presence/absence data were assessed using Veech’s pairwise co-occurrence approach [18] via the cooccur package [19]. We excluded antimicrobial resistance genes (ARGs) whose total abundance was lower than 0.5% [20]. This method employs a probabilistic approach to determine which AMR gene pairs co-occur more (positive co-occurrence) or less often (negative co-occurrence) than is likely by chance. Networks of AMR gene pairs considered to have significant positive co-occurrence and were commonly found were visualised using the igraph package [21].

### Beta diversity

To test for differences in AMR gene assemblage between host populations, permutational multivariate analysis of variance (PERMANOVA) on the Jaccard Distance Matrix of ARG composition was performed using the adonis function in the vegan package with 999 permutations. In the adonis analysis, ‘host group’ was used as a fixed factor and a strata argument set to ‘household ID’ to constrain randomizations within each household. Principal coordinate analysis (PCoA) was used to ordinate the Jaccard Distance Matrix in the vegan package and visualised using ggplot2 [22]. Confidence ellipses were drawn around isolates from each host group, using a 95% confidence interval.

### Modelling AMR gene exchange at the household level

To investigate the potential drivers of AMR gene carriage in humans, we used a zero-inflated Poisson general linear mixed effects model (GLMM) with counts of the individual AMR genes (also referred to as AMR gene length) in each of the isolate aggregated at the antibiotic class level as the dependent variable. Risk factors analysed included the following: household size (persons in a household as a function of household area), disposal practices (manure disposed in the household compound or outside), toilet sharing, kinds of livestock kept in the household (small livestock only, large livestock + / − small livestock and no livestock). Moreover, for households that kept livestock, a separate zero-inflated Poisson GLMM was fitted to investigate the influence of household size and manure disposal practices (manure disposed in the household compound or outside) on AMR gene length.

Analyses were performed using the glmmTMB package [23] and significance was determined using the Wald *χ*^2^ tests via package car [24]. The top-ranked model was selected using minimal Akaike Information Criterion using the package MuMIn [25]. To account for the nested nature of our sampling design, household site (*n* = 99) was included as a random effect. We plotted the diagnostic plots of the zero-inflated Poisson model, including random effects, to check that the model assumptions were not violated using the package DHARMa [26]. All analysis was conducted in R v4.0.3.

## Results

A total of 1316 isolates were sequenced of which 311 were (23.6%) from humans and 606 (46.1%) from 13 different species of livestock primarily composed of poultry (*n* = 324), goats and sheep (*n* = 134), cattle (*n* = 61), pigs (*n* = 49) and rabbits (*n* = 38). In addition, 399 (30.3%) of the genomes were obtained from 63 wildlife species, primarily comprised of wild birds (*n* = 245), rodents (*n* = 130), bats (*n* = 20) and primates (*n* = 4) (Additional file 1: Table S1).

### Distribution of antimicrobial resistance genes and mutations across human and animal populations

Analysis of the 1316 genomes identified 56 unique ARGs, along with 13 point mutations (seven in *parC* region, four *gyrA* and two *parE*). In total, these genes and mutations confer resistance to a total of nine antibiotic classes (Additional file 1: Table S2). Nearly half (607 isolates, 46.2%) of the *E. coli* isolates analysed were pan-susceptible, where no AMR genes or mutations were detected. A significantly larger proportion of pan-susceptible isolates was recovered from wildlife (225 isolates, 56.4%) than humans (100 isolates, 32.2%) and livestock (282 isolates, 46.5%) (*p* < 0.001, Kruskal–Wallis, Bonferroni corrected). Sixty percent (*n* = 100), 45.5% (*n* = 282) and 36.6% (*n* = 225) of human, livestock and wildlife isolates respectively carried AMR mechanisms known to confer resistance to three or more antibiotic classes (or multi-drug resistance carriage), representing an overall carriage of 46.3% (*n* = 609) (Additional file 1: Figure S2).

**Fig. 1:**
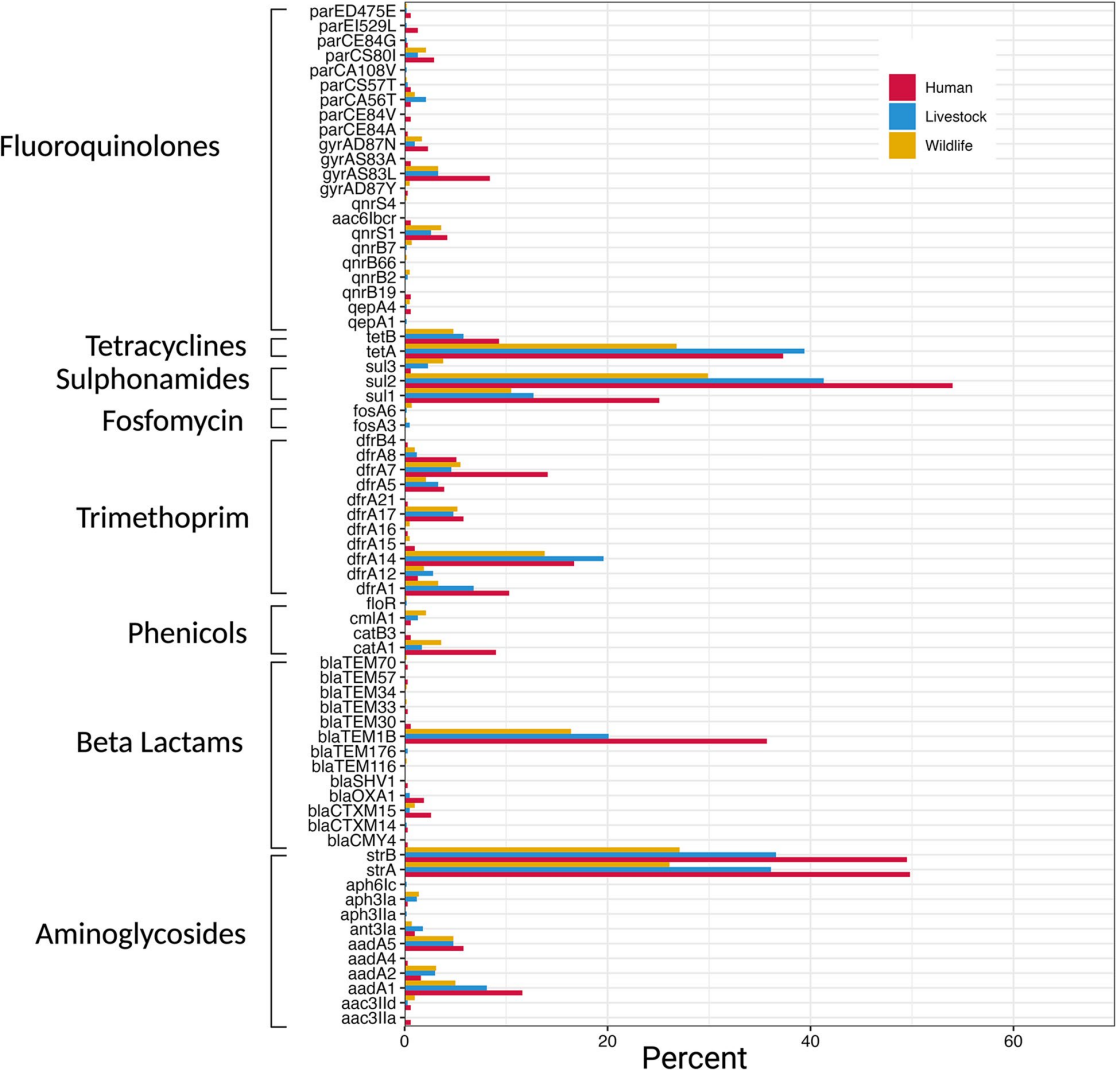
Prevalence of 56 AMR genes and 13 AMR-conferring point mutations in 311 human, 606 livestock and 399 wildlife *E. coli* isolates collected from the 99 households in Nairobi, Kenya (2015–2016)

Across all isolates, the most common AMR genes were *sul2* (41.3%), *strA* (36.8%), *strB* (37.2%), *tetA* (35.6%) and *bla*_*TEM-1B*_ (23%) conferring resistance to sulphonamides, aminoglycosides, tetracyclines and beta-lactams respectively (Fig. 1). Of note, 365 (27%) isolates harboured at least one of seventeen different extended-spectrum β-lactamase (ESBL) genes. Eight human, three livestock (two ducks and one pig) and four wildlife (three wild birds and one rodent) isolates carried the clinically relevant *bla*_*CTX-M-15*_ gene.

The distributions of most (61/69, 88.4%) of the AMR genes and mutations did not significantly differ between the host groups. Nevertheless, eight ARGs (*bla*_*TEM-1B*_, *catA1*, *dfrA7*, *dfrA8*, *strA*, *strB*, *sul1*, *sul2*) were significantly more common in human isolates than in livestock and wildlife, whilst *strA*, *strB* and *sul2* genes were detected at higher rates in livestock isolates than in wildlife (*p* < 0.05, Kruskal–Wallis, Bonferroni corrected).

Probabilistic modelling of acquired AMR genes co-occurrence revealed 158 (41.7%, *n* = 378) pairs of AMR genes co-occurring significantly more frequently than expected, 22 (5.8%) pairs of AMR genes co-occurring significantly less frequently than expected and *n* = 198 (52.3%) random AMR genes associations (observed frequency of co-occurrence does not significantly depart from expected) (Additional file 1: Figure S3). The most common co-occurring gene combination comprised of *strA*, *strB* and *sul2*. This combination of three genes was present in 465 isolates (35.3%, *n* = 1316) either singly or in combination with other ARGs and was significantly more common in humans than animal hosts (*χ*^2^ = 38.2, *p* < 0.0001, chi-squared test). Further, the *strA-strB-sul2* gene combination significantly co-occurred with *tetA* in 334 (25.1%) isolates and with both *tetA* and *bla*_*TEM-1B*_ in 190 (14.4%) isolates (*p* < 0.001, chi-squared test) (Additional file 1: Figure S4).

### Analysis of alpha and beta diversity of AMR genes

**Fig. 2:**
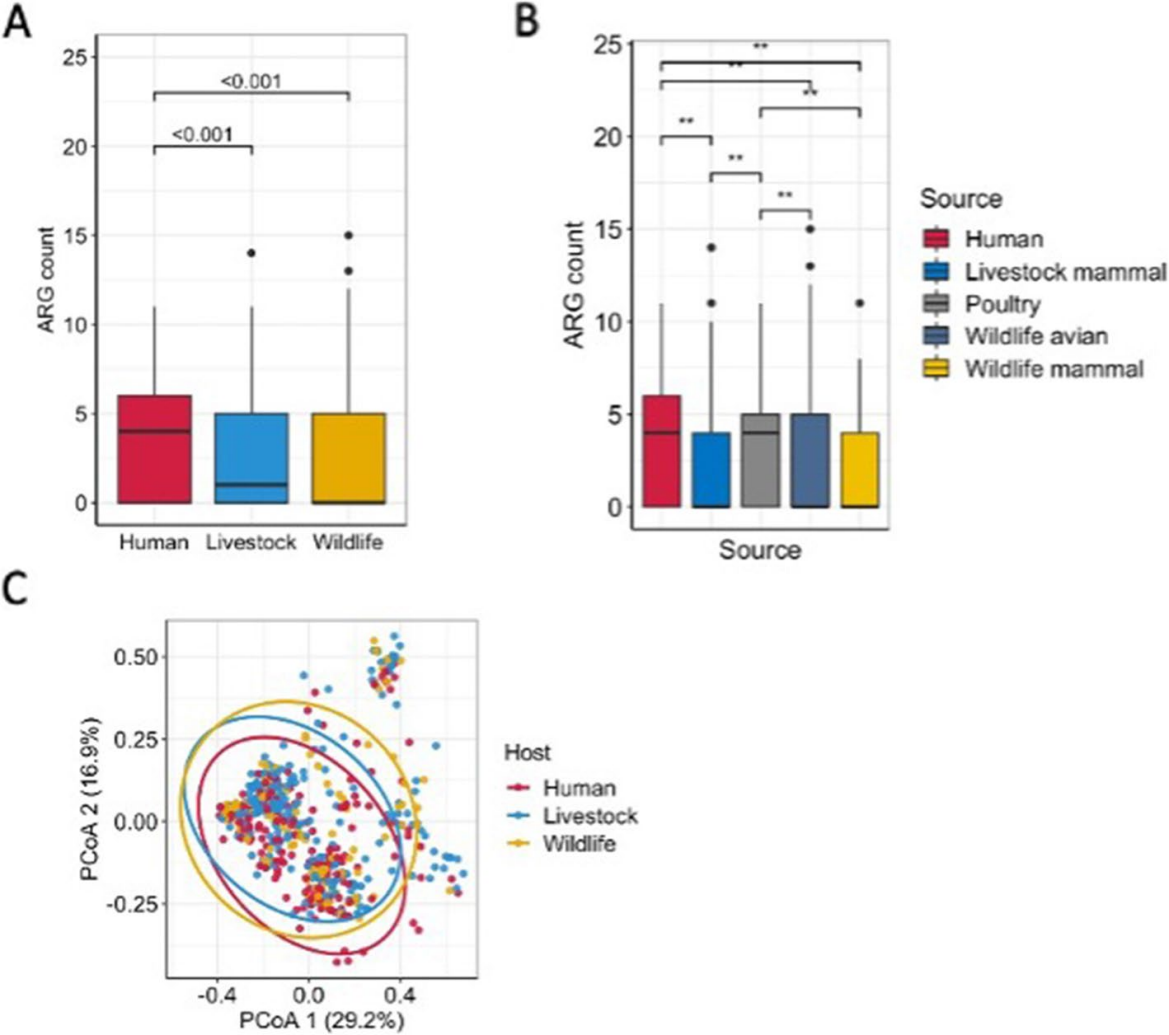
**a**–**c** Number of AMR genes per isolate in **a** humans, livestock and wildlife; **b** humans and the different animal host groups. Data are shown via the interquartile ranges (IQRs), median as a black horizontal line and the whiskers extending up to the most extreme points within 1.5 × the IQR; outliers are represented as dots. *p* values were calculated using the Wilcoxon test and Kruskal–Wallis. **c** Principal coordinate analysis (PCoA) of AMR genes from all host groups based on the Jaccard distance matrix. *p* values of beta diversity were calculated with PERMANOVA by 999 permutations. Ellipsoids represent a 95% confidence interval surrounding each host group

**Fig. 3:**
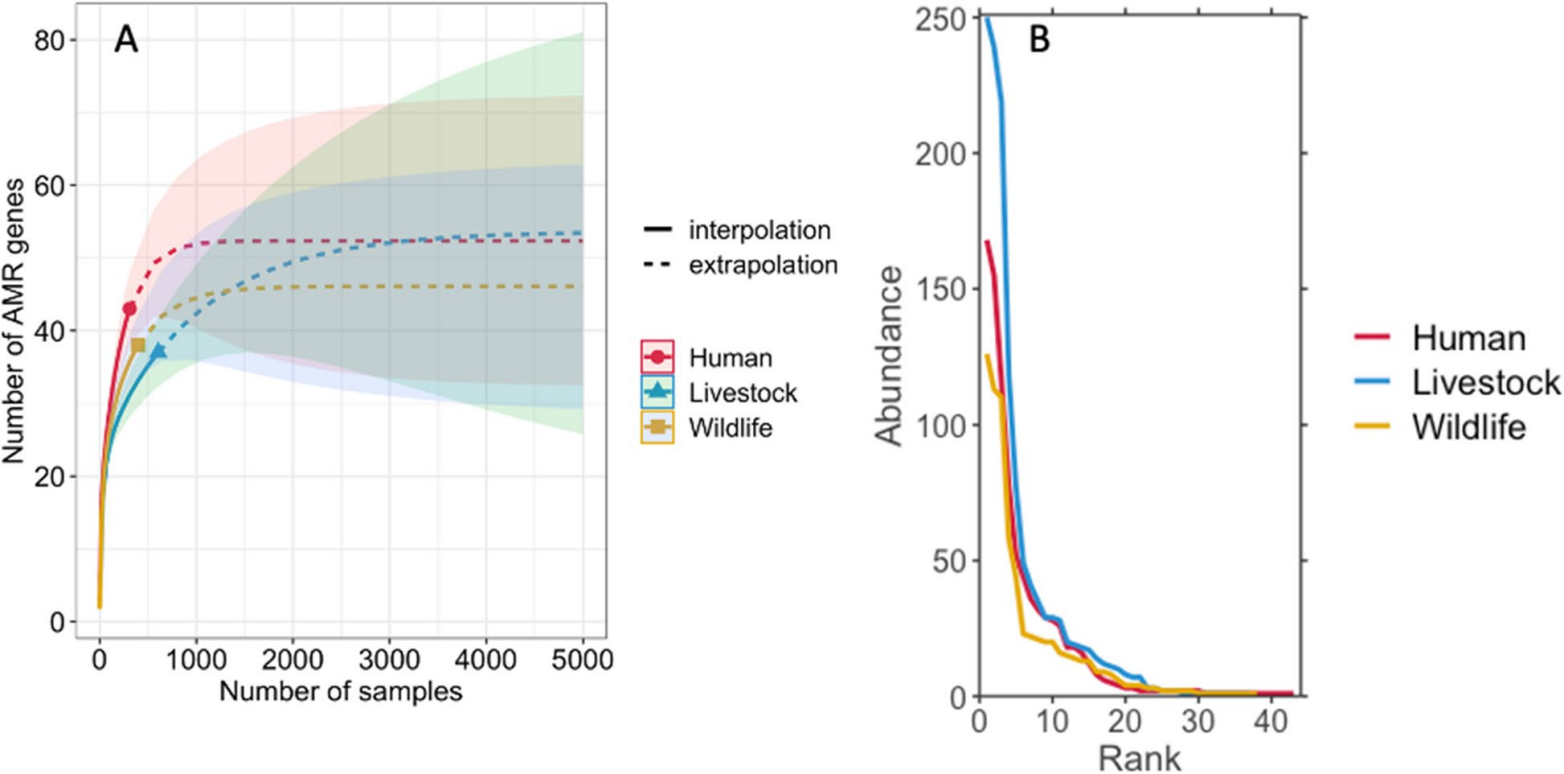
**a** Sample size-based rarefaction and extrapolation sampling curves for acquired AMR genes in our dataset. Solid lines (red—human, blue—livestock and gold—wildlife) are the rarefaction curves. Dotted lines indicate Chao2 prediction of asymptotic richness by number of samples (predicted AMR gene numbers were 53.8, 54 and 47 for humans, livestock and wildlife, respectively). Shading indicates 95% confidence intervals and solid circle, box and triangle shapes are the reference samples and the observed number of AMR genes (44, 38 and 39 for humans, livestock, and wildlife, respectively). **b** Rank abundance curve of the acquired AMR genes detected in humans (red line), livestock (blue line) and wildlife (gold line)

Human isolates had more acquired AMR genes than livestock and wildlife isolates (median 4 vs. 1 vs. 0; *p* < 0.0001) (Fig. 2a). Similarly, human isolates had more acquired AMR genes than all livestock and wildlife groups with the exception of poultry isolates (*p* < 0.001, Kruskal–Wallis, Bonferroni corrected). Poultry isolates had higher AMR carriage than ‘livestock mammals’, ‘wildlife avian’ and ‘wildlife mammals’ isolates (*p* < 0.001, Kruskal–Wallis, Bonferroni corrected) (Fig. 2b). This pattern was consistent with both Simpson and Shannon alpha-diversity indexes (Additional file 1: Figure S5). Rarefaction analyses showed that 83%, 69% and 82% of the resistance gene diversity that could be sampled from human, livestock and wildlife populations respectively was captured by our sample size (Fig. 3a and Additional file 1 Table S3). Additionally, rank abundance curves of the observed AMR gene diversity indicated that only a few of these genes dominated the community, whilst most occurring rarely (Fig. 3b).

However, these differences in the relative abundance and alpha diversity of AMR genes were not reflected in the overall AMR gene community composition when compared between host groups via beta diversity, which did not significantly differ between human and animal host groups (Fig. 2c, Adonis, *p* > 0.05).

### Drivers of AMR gene carriage at the household level

**Fig. 4:**
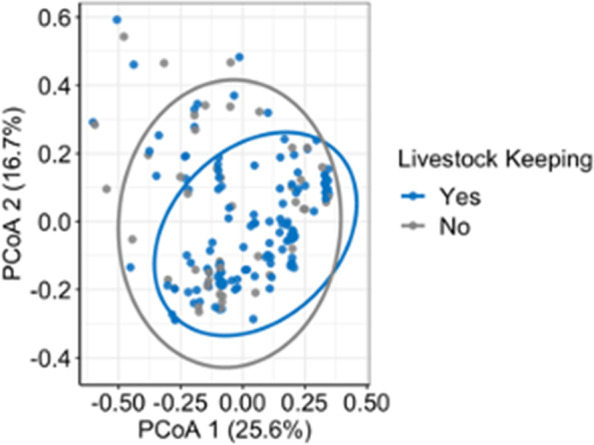
Principal coordinate analysis (PCoA) of the Jaccard distance matrix describing AMR gene assemblage in humans with respect to the livestock-keeping status. *p* values of beta diversity were calculated with PERMANOVA by 999 permutations. Ellipsoids represent a 95% confidence interval surrounding each group. Blue circle and points correspond to people who kept livestock in the household, and grey corresponds to people without livestock

Next, we assessed whether livestock keeping, and livestock keeping practices, influenced AMR gene carriage in humans. First, we analysed whether AMR gene composition in isolates from humans varied with respect to their livestock-keeping status. Permutational multivariate analysis of variance (PERMANOVA) analysis showed that the AMR gene composition of humans keeping livestock did not differ from that of humans not keeping livestock (adonis *R*^2^ = 0.01, *p* = 0.98, Fig. 4). Consistently, the AMR gene composition of humans did not vary depending on the kind of livestock kept in the household (Adonis *R*^2^ < 0.01, *p* = 0.97).

**Fig. 5:**
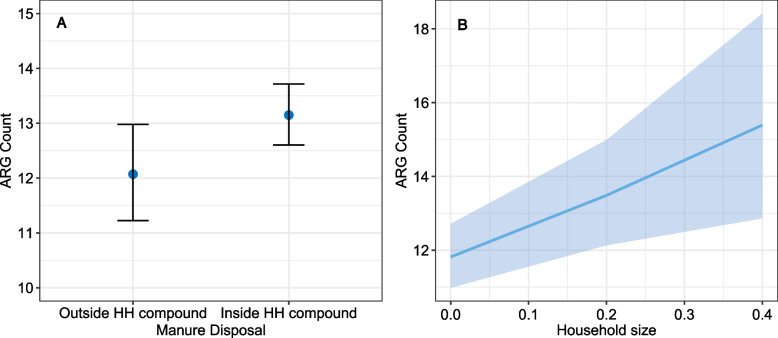
**a**, **b** Fit of generalised linear mixed effects models relating AMR carriage in humans to **a** manure disposal practices and **b** household size (persons in a household as a function of household area). The 95% confidence interval is represented by error bars and the transparent band, respectively

Results from mixed-effects models corroborated this result and did not support the hypothesis that the presence of livestock (small or large + / − small) influences the risk of human AMR gene carriage (*p* > 0.05) (model 1, Table 1). The impact of livestock is, however, moderated by manure management practices: human AMR gene carriage is significantly higher if manure was kept inside the household perimeter compared to disposing of externally (OR = 1.4, *p* = 0.03, 95% CI [1.02–1.8]) (model 2, Table 1; Fig. 5a). Furthermore, our model indicated that human carriage of AMR genes was significantly associated with increasing household size (OR = 5.77, *p* = 0.003, 95% CI [1.85–18.04]) (model 2, Table 1; Fig. 5b).

**Table 1:** Results of zero-inflated Poisson generalised linear mixed effects models investigating household risk factors for AMR gene carriage in humans at the household level. Households not keeping livestock used as the reference level in model 1

	Odds ratio	Confidence interval	*p* value
Model 1: AMR gene length, humans in all households			
Large livestock (+ / − small livestock)	0.83	0.55–1.25	0.37
Small livestock only	0.85	0.60–1.22	0.39
Model 2: AMR gene length, humans in livestock-keeping households only			
Household size	5.77	1.85–18.04	**0**.**003**
Manure in household	1.4	1.02–1.8	**0**.**03**

## Discussion

This study combines epidemiological approaches and genomic analyses to understand AMR on systematically collected *E. coli* isolates obtained from sympatric human and animal populations in a rapidly developing urban landscape. We showed that whilst patterns of carriage, diversity and assemblages of resistance mechanisms in *E. coli* are generally similar across human, livestock and wildlife hosts, some differences exist across the three groups, attributable to host-specific drivers. Our analyses indicate that differences in carriage of AMR mechanisms in humans were not associated with livestock keeping per se but instead associated with livestock-keeping practices that influence environmental contamination such as manure disposal practices, and household size.

Nearly half of all isolates (46.3%) carried AMR gene mechanisms known to confer resistance to three or more antibiotic classes. The most common resistance mechanisms encoded in the mapped genomes were associated with resistance to sulphonamides, aminoglycosides, tetracyclines and beta-lactams, in agreement with our earlier studies exploring phenotypic patterns expressed by bacteria [5, 27] and in previous studies in Africa [28, 29]. Most AMR genes (88.5%) were found in similar proportions in both human, livestock and wildlife isolates, highlighting their ubiquitous distribution in both reservoirs, while a minority (12.5%) were significantly more common in human than in animal isolates. Previous studies in similar settings of Thailand [30], Nigeria [31] and Vietnam [32] that compared isolates from humans and livestock indicated the ubiquitous distribution of AMR across host niches. There was a greater abundance and diversity of acquired AMR genes in humans and poultry than in other livestock and wildlife groups. This finding is possibly attributable to the high antibiotic usage in humans and the rapidly growing intensive poultry farming system [33] or the frequent contact between humans and poultry, although reliable data are lacking. We detected ESBL carriage in 27% of our isolates, with three wild birds and one rodent carrying the highly clinically relevant *bla*_*CTX-M-15*_ gene. Several studies conducted mostly in *E. coli* have identified AMR genes including ESBL-carrying bacteria in numerous species of wildlife dominated by wild birds [34–37]. Community settings in Nairobi—potentially contaminated with animal waste and human sewage from human settlements and hospital settings—could act as pools of AMR to which wildlife are exposed. That urban wildlife likely represent a net sink for AMR genes was further supported by the fact that they contained significantly more pan-susceptible isolates than human or livestock. These results highlight the need for ecosystem-wide surveillance of AMR in urban areas. The finding that our sampling effort captured the majority of AMR gene diversity in the study populations highlights the viability of future surveillance studies of ARGs with similar sample sizes and epidemiologically structured designs. However, to increase the likelihood of detecting new AMRs mechanisms in these populations, especially in animal hosts, additional sampling and longitudinal effort, potentially combined with metagenomic sequencing, are needed.

We found that overall AMR community composition overlapped among host groups and that AMR gene communities of humans keeping livestock were not different from those of people who did not keep livestock. In complex urban interfaces such as Nairobi, humans, livestock and wildlife are linked in many ways, including direct contact, consumption of livestock products by humans and shared contaminated environments for example through human sewage and manure from livestock [38]. These results corroborate earlier phylogenetic findings from the same study population that indicated inter-host resistome and pangenome similarity irrespective of genomic relatedness [9]. This connectedness presumably allows a circular flow of AMR bacteria and AMR determinants across host groups, potentially explaining the observed overlap [32, 39]. It might also be speculated that selection for AMR due to overlapping patterns of antibiotic use in both human and livestock populations explains the similarity in AMR gene communities. We could not test these hypotheses using our data; a combination of rigorous epidemiological designs, involving longitudinal studies with repeated sampling on the same individual or animal, and high-resolution phylogeographic methods are still required to understand the exact direction and frequency of AMR transmission [8]. Importantly, our data do not deny a potential role for livestock in the propagation of AMR determinants in this system but suggest that this role, should it be significant, is through the dissemination of livestock (and human) waste in a broader environment, and that individual host contact with that broader environment determines the individual level risk of acquiring AMR infection.

Our analysis suggests that the potential for AMR transmission across human-livestock interfaces is greatest when manure is poorly disposed of, and when household size is larger. Environmental exposure to manure—a common soil fertiliser in crop farming—provides an ideal environment for the amplification and persistence of AMR determinants [40, 41] and can have adverse effects on the health of ecosystems and humans. Similarly, overcrowded urban environments typically associated with inadequate hygiene also contribute to the dispersal of AMR bacteria and resistance determinants. Infrastructural improvements focused on water, sanitation and hygiene and biosecurity improvements such as pre-treatment of manure are needed to de-risk ecosystems and food chains.

By only sequencing a single *E. coli* isolate from each host, the within-host genetic diversity of *E. coli* was not considered in this study. Recent studies have revealed that in some bacteria there is considerable within-host diversity and AMR gene diversity [42]. In this study, the decision to sequence a single isolate from each host was made as a cost-based trade-off between the depth of sampling *E. coli* genetic diversity within each individual host and the number of unique individuals from which samples could be included. Future research could benefit from the recent development and use of metagenomics to characterise the abundance, diversity and structure of acquired resistomes [43].

Findings from this study fill important data gaps concerning the frequency of AMR genes in human and animal reservoirs in LMICs and provide further support for sustainable application of WGS and genomic epidemiology in contributing to surveillance efforts of AMR mechanisms circulating in different ecosystems [44, 45]. Further studies are required to understand whether our findings will be reproduced in other geographical areas, and to investigate whether the noticed AMR carriage in this study is transient or a more permanent colonisation [46].

## Conclusions

In conclusion, we present a genomic study of *E. coli* in sympatric human and animal populations in a rapidly developing urban setting. By stepping outside of the ‘blame game’ of livestock, and human health, our study, applying a ‘One Health’ approach, demonstrates that AMR genes conferring resistance to critically important antimicrobials for both human and veterinary medicine are widespread in humans, livestock and wildlife hosts. We found that human and animal AMR gene profiles were similar but did not detect an association between livestock keeping and AMR gene numbers or composition in humans. Instead, the impact of keeping livestock on human AMR carriage was mediated by practices associated with livestock keeping, namely the presence or absence of animal manure in the household and with household size. Taken together, our study serves as a model for the targeted sampling to harness the power of whole genome sequencing for understanding the epidemiology of AMR across developing urban landscapes, a key in developing effective strategies to reduce the development and spread of such resistance in the future.

## Supplementary Information

**Additional file 1.** 

## Data Availability

Whole-genome sequences used in this study are available under the BioProjects with accession numbers PRJEB32607 and PRJEB41827. Data that support the findings of this study are available in the Dryad Digital Repository with the identifiers 10.5061/dryad.qnk98sfkf (47).

## Abbreviations

AMR: Antimicrobial Resistant
ARGs: Antimicrobial Resistance Genes
PERMANOVA: Permutational Multivariate Analysis of Variance
PCoA: Principal Coordinate Analysis
GLMM: Poisson General Linear Mixed Effects Model
IQR: Interquartile range
OR: Odds Ratio
ESBL: Extended-Spectrum Beta-Lactamase

## References

[1] Murray CJL, Ikuta KS, Sharara F, Swetschinski L, Robles Aguilar G, Gray A, et al. Global burden of bacterial antimicrobial resistance in 2019: a systematic analysis. Lancet. 10.1016/S0140-6736(21)02724-0 PMC8841637 35065702

[2] Tang KL, Caffrey NP, Nóbrega DB, Cork SC, Ronksley PE, Barkema HW (2017). Restricting the use of antibiotics in food-producing animals and its associations with antibiotic resistance in food-producing animals and human beings: a systematic review and meta-analysis. Lancet Planet Health.

[3] Woolhouse M, Ward M, van Bunnik B, Farrar J. Antimicrobial resistance in humans, livestock and the wider environment. Philos Trans R Soc Lond B Biol Sci. 2015;370(1670):20140083. 10.1098/rstb.2014.0083 PMC4424433 25918441

[4] Vittecoq M, Godreuil S, Prugnolle F, Durand P, Brazier L, Renaud N (2016). Antimicrobial resistance in wildlife. J Appl Ecol.

[5] Hassell JM, Ward MJ, Muloi D, Bettridge JM, Robinson TP, Kariuki S (2019). Clinically relevant antimicrobial resistance at the wildlife–livestock–human interface in Nairobi: an epidemiological study. Lancet Planet Health.

[6] Kern W, Rieg S (2020). Burden of bacterial bloodstream infection—a brief update on epidemiology and significance of multidrug-resistant pathogens. Clin Microbiol Infect.

[7] Muloi D, Ward MJ, Pedersen AB, Fevre EM, Woolhouse MEJ, van Bunnik BAD (2018). Are food animals responsible for transfer of antimicrobial-resistant Escherichia coli or their resistance determinants to human populations? A systematic review. Foodborne Pathog Dis.

[8] Wee BA, Muloi DM, van Bunnik BAD (2020). Quantifying the transmission of antimicrobial resistance at the human and livestock interface with genomics. Clin Microbiol Infect.

[9] Muloi DM, Wee BA, McClean DMH, Ward MJ, Pankhurst L, Phan H, et al. Population genomics of Escherichia coli in livestock-keeping households across a rapidly developing urban landscape. Nat Microbiol. 2022;7(4):581-9. 10.1038/s41564-022-01079-y PMC8975746 35288654

[10] Bettridge JM, Robinson TR, Hassell JM, Kariuki S, Ward MJ, Woolhouse MEJ, et al., editors. An epidemiologically structured sampling strategy to capture bacterial diversity in a changing urban environment. Proceedings of the Society for Veterinary Epidemiology and Preventive Medicine; 2017; United Kingdom.

[11] Bharat A, Petkau A, Avery BP, Chen JC, Folster JP, Carson CA (2022). Correlation between phenotypic and in silico detection of antimicrobial resistance in Salmonella enterica in Canada using Staramr. Microorganisms.

[12] Zankari E, Hasman H, Cosentino S, Vestergaard M, Rasmussen S, Lund O (2012). Identification of acquired antimicrobial resistance genes. J Antimicrob Chemother.

[13] Zankari E, Allesøe R, Joensen KG, Cavaco LM, Lund O, Aarestrup FM (2017). PointFinder: a novel web tool for WGS-based detection of antimicrobial resistance associated with chromosomal point mutations in bacterial pathogens. J Antimicrob Chemother.

[14] Oksanen J, Blanchet FG, Friendly M, Kindt R, Legendre P, McGlinn D, et al. vegan: Community Ecology Package. R package version 2.5–7. 2020.

[15] Hughes JB, Hellmann JJ, Ricketts TH, Bohannan BJM (2001). Counting the uncountable: statistical approaches to estimating microbial diversity. Appl Environ Microbiol.

[16] Hsieh T, Ma K, Chao A (2016). iNEXT: an R package for rarefaction and extrapolation of species diversity (H ill numbers). Methods Ecol Evol.

[17] Chao A, Jost L (2012). Coverage-based rarefaction and extrapolation: standardizing samples by completeness rather than size. Ecology.

[18] Veech JA (2013). A probabilistic model for analysing species co-occurrence. Glob Ecol Biogeogr.

[19] Griffith DM, Veech JA, Marsh CJ (2016). Cooccur: probabilistic species co-occurrence analysis in R. J Stat Softw.

[20] Saiz H, Gómez-Gardeñes J, Borda JP, Maestre FT (2018). The structure of plant spatial association networks is linked to plant diversity in global drylands. J Ecol.

[21] Csardi G, Nepusz T (2006). The igraph software package for complex network research. InterJournal, Complex Syst.

[22] Wickham H. ggplot2: Elegant Graphics for Data Analysis. New York: Springer-Verlag; 2016.

[23] Magnusson A, Skaug H, Nielsen A, Berg C, Kristensen K, Maechler M, et al. glmmTMB: generalized linear mixed models using Template Model Builder. R package version 0.1. 0. 2017.

[24] Fox J, Weisberg S, Adler D, Bates D, Baud-Bovy G, Ellison S (2012). Package ‘car’.

[25] Barton K, Barton MK (2015). Package ‘mumin’. Version.

[26] Hartig F. DHARMa: residual diagnostics for hierarchical (multi-level/mixed) regression models. R package version 03. 2020;3.

[27] Muloi D, Kiiru J, Ward MJ, Hassell JM, Bettridge JM, Robinson TP (2019). Epidemiology of antimicrobial-resistant Escherichia coli carriage in sympatric humans and livestock in a rapidly urbanizing city. Int J Antimicrob Agents.

[28] Subbiah M, Caudell MA, Mair C, Davis MA, Matthews L, Quinlan RJ (2020). Antimicrobial resistant enteric bacteria are widely distributed amongst people, animals and the environment in Tanzania. Nat Commun.

[29] Ingle DJ, Levine MM, Kotloff KL, Holt KE, Robins-Browne RM (2018). Dynamics of antimicrobial resistance in intestinal Escherichia coli from children in community settings in South Asia and sub-Saharan Africa. Nat Microbiol.

[30] Hickman RA, Leangapichart T, Lunha K, Jiwakanon J, Angkititrakul S, Magnusson U, et al. Exploring the Antibiotic Resistance Burden in Livestock, Livestock Handlers and Their Non-Livestock Handling Contacts: A One Health Perspective. Front Microbiol. 2021;12:651461. 10.3389/fmicb.2021.651461 PMC8093850 33959112

[31] Aworh MK, Kwaga J, Okolocha E, Harden L, Hull D, Hendriksen RS (2020). Extended-spectrum ß-lactamase-producing Escherichia coli among humans, chickens and poultry environments in Abuja, Nigeria. One Health Outlook.

[32] Nguyen VT, Jamrozy D, Matamoros S, Carrique-Mas JJ, Ho HM, Thai QH (2019). Limited contribution of non-intensive chicken farming to ESBL-producing Escherichia coli colonization in humans in Vietnam: an epidemiological and genomic analysis. J Antimicrob Chemother.

[33] Muloi D, Fevre EM, Bettridge J, Rono R, Ong’are D, Hassell JM, et al. A cross-sectional survey of practices and knowledge among antibiotic retailers in Nairobi, Kenya. J Glob Health. 2019;9:020412. 10.7189/jogh.09.020412 PMC6708591 31489183

[34] Alcalá L, Alonso CA, Simón C, González-Esteban C, Orós J, Rezusta A (2016). Wild birds, frequent carriers of extended-spectrum β-lactamase (ESBL) producing Escherichia coli of CTX-M and SHV-12 types. Microb Ecol.

[35] Ben Yahia H, Ben Sallem R, Tayh G, Klibi N, Ben Amor I, Gharsa H (2018). Detection of CTX-M-15 harboring Escherichia coli isolated from wild birds in Tunisia. BMC Microbiol.

[36] Schaufler K, Nowak K, Düx A, Semmler T, Villa L, Kourouma L, et al. Clinically Relevant ESBL-Producing K. pneumoniae ST307 and E. coli ST38 in an Urban West African Rat Population. Front Microbiol. 2018;9:150. 10.3389/fmicb.2018.00150 PMC5812336 29479341

[37] Fashae K, Engelmann I, Monecke S, Braun SD, Ehricht R (2021). Molecular characterisation of extended-spectrum ß-lactamase producing Escherichia coli in wild birds and cattle, Ibadan, Nigeria. BMC Vet Res.

[38] Nadimpalli ML, Marks SJ, Montealegre MC, Gilman RH, Pajuelo MJ, Saito M (2020). Urban informal settlements as hotspots of antimicrobial resistance and the need to curb environmental transmission. Nat Microbiol.

[39] Nadimpalli ML, Stegger M, Viau R, Yith V, de Lauzanne A, Sem N, et al. Leakiness at the human-animal interface in Southeast Asia and implications for the spread of antibiotic resistance. bioRxiv. 2021:2021.03.15.435134.

[40] Udikovic-Kolic N, Wichmann F, Broderick NA, Handelsman J (2014). Bloom of resident antibiotic-resistant bacteria in soil following manure fertilization. Proc Natl Acad Sci.

[41] Graham DW, Knapp CW, Christensen BT, McCluskey S, Dolfing J (2016). Appearance of β-lactam resistance genes in agricultural soils and clinical isolates over the 20th century. Sci Rep.

[42] Stoesser N, Sheppard AE, Moore CE, Golubchik T, Parry CM, Nget P (2015). Extensive within-host diversity in fecally carried extended-spectrum-beta-lactamase-producing Escherichia coli isolates: implications for transmission analyses. J Clin Microbiol.

[43] Munk P, Knudsen BE, Lukjancenko O, Duarte ASR, Van Gompel L, Luiken REC (2018). Abundance and diversity of the faecal resistome in slaughter pigs and broilers in nine European countries. Nat Microbiol.

[44] Baker S, Thomson N, Weill F-X, Holt KE (2018). Genomic insights into the emergence and spread of antimicrobial-resistant bacterial pathogens. Science.

[45] WHO. GLASS whole-genome sequencing for surveillance of antimicrobial resistance. 2020. World Health Organization; 2020.

[46] Sun J, Yang M, Sreevatsan S, Bender JB, Singer RS, Knutson TP (2017). Longitudinal study of Staphylococcus aureus colonization and infection in a cohort of swine veterinarians in the United States. BMC Infect Dis.

[47] Dishon M, et al. Genomic epidemiology of Escherichia coli: antimicrobial resistance through a One Health lens in sympatric humans, livestock and peri-domestic wildlife in Nairobi, Kenya, Dryad: Dataset; 2022. 10.5061/dryad.qnk98sfkf. 10.1186/s12916-022-02677-7 PMC9730568 36482440

