# Supplementary material for: Genomic epidemiology of Escherichia coli: antimicrobial resistance through a One Health lens in sympatric humans, livestock and peri-domestic wildlife in Nairobi, Kenya
Source: BMC Med. 2022 Dec 8;20:471. doi: 10.1186/s12916-022-02677-7 (PMC9730568; doi:10.1186/s12916-022-02677-7)
Supplement: Supplementary file 1 — Additional file 1. [file 12916_2022_2677_MOESM1_ESM.docx]

**Additional File 1**

**For**

**Genomic epidemiology of *Escherichia coli*: antimicrobial resistance through a One Health lens in sympatric humans, livestock and peri-domestic wildlife in Nairobi, Kenya**

Dishon M. Muloi^1,2,4*^, James M. Hassell^3,4^, Bryan A. Wee^1^, Melissa J. Ward ^2,5,11^_,_ Judy M. Bettridge ^3, 4^, Velma Kivali^4^, Alice Kiyong’a^4^, Christine Ndinda^4^, Nduhiu Gitahi^8^, Tom Ouko^7^, Titus Imboma^10^, James Akoko^4^, Maurice K. Murungi ^4^, Samuel M. Njoroge^7^, Patrick Muinde^4^, Lorren Alumasa ^4^, Titus Kaitho^10^, Fredrick Amanya^4^, Allan Ogendo^4^, Bram A.D. van Bunnik^1^, John Kiiru^7^, Timothy P. Robinson^6^, Erastus K. Kang’ethe^8^, Samuel Kariuki^7^_,_ Amy B Pedersen^2, 9^, Eric M. Fèvre^3, 4+^, Mark E. J. Woolhouse^1, 2*+^

* Corresponding author

^+^These authors contributed equally

**This file includes:**

**Table S1.** Number and percentage of humans, individual wildlife and livestock sampled as part of the study.

**Table S2.** Summary of AMR genes and point mutations classified by host type.

**Table S3.** Asymptotic estimates for the total number of AMR genes in humans, livestock and wildlife isolates and estimated sampling effort.

**Figure S1.** Geographical distribution of selected sublocations within the city of Nairobi.

**Figure S2 (A-C)**. Histograms illustrating the number of antibiotic classes to which A) Human, B) Livestock, C) Wildlife E. coli isolates were detected as having AMR associated mechanisms.

**Figure S3**. Heatmap of pairwise positive, negative and random associations calculated according to a probability co‐occurrence model for acquired AMR genes found in 0.5% of the isolates.

**Figure S4.** Network analysis revealing the AMR co-occurrence patterns among human, livestock and wildlife E.coli isolates.

**Figure S5.** Box-plots of A) Shannon diversity and B) Simpson index alpha diversity indices of acquired AMR genes.

**Supplementary methods**

**Data Collection**

**Sampling of humans:** In the household, a questionnaire was administered to the household head (or a nominated person) to collect data on socio-economic variable (e.g. income), livestock ownership (e.g., diversity and number of livestock kept), management practices (e.g., manure and garbage disposal practices), household composition (e.g., number of occupants) and household area. Following informed consent, all persons were asked to provide a faecal sample and answer questionnaires on: their age, gender and occupation, food consumption, income, and medical history. The number of persons recruited per household varied by wealth and ranged between one and 19 persons.

**Sampling of livestock:** Rectal swabs were collected from the representative livestock species in a household. In total up to eleven different species of livestock (cattle, pigs, sheep, goats, rabbits, chickens, ducks, geese, turkeys, guinea fowl and pigeons) were recruited and sampled over the course of the study (table s1). The distribution of livestock between neighbourhood classes varied according to species. Chickens were the most common species encountered, kept by 83% of the 66 livestock-keeping households; these along with goats, rabbits and other poultry types were distributed relatively evenly across all neighbourhood classes. However, cattle and sheep were found almost exclusively in either the very wealthy areas, the very poor areas, or the areas on the eastern and western periphery of the city. The distribution of pigs was similar, except that they were not found in the higher wealth groups, although one pig-keeper in a dense new-build area (wealth group 5) was recruited.

**Sampling of wildlife:** Rodents, bats, birds and non-human primates were sampled. Rodents were trapped using medium-sized (23 cm x 7.5 cm x 9 cm) Sherman live traps (H. B. Sherman Traps Inc., Tallahassee, FL) or Victor lethal traps (Woodstream Corp., Lititz, PA) that were baited with dried fish, placed against walls throughout the household and livestock keeping facilities, and left in place for three nights. Traps were set in each household for all trapping nights and checked daily. Mist nets were set at dawn to trap birds, with nets being positioned outside the house and around livestock keeping facilities. For household compounds in which bat activity was deemed likely (as judged based on the presence of fruiting trees and/or ‘flyways’), mist nets were set at dusk and monitored for two hours. Where household members reported frequent sightings of non-human primates, wire-mesh live-capture traps were pre-baited with bananas for a minimum of three days. Traps were then set, and monitored regularly for a maximum of three days. Due to large variation in the size of household compounds, trapping effort (i.e. number of traps/mist nets placed per trapping session) was maintained such that it was proportional to the size of the household compound.

Once caught, all birds, and all but two bats caught per trapping session, were live-sampled in the field under manual restraint, before being released unharmed. All live rodents (except for individuals belonging to the genus Cricetomys, which were live-sampled under anaesthesia) and up to two bats caught per trapping session were transferred back to a biosafety level three (BSL3) laboratory at ILRI. Trapped rodents and bats were placed in containers that were resistant to escape, provided adequate ventilation and protection from the elements. Holding containers were transported via project vehicles that were decontaminated after use. At the labortaory, the animals were humanely euthanised by cardiac puncture under isoflurane anaesthesia and a full post-mortem examination then performed under enhanced BSL2 conditions, with fresh faeces being collected from the rectum. Rodents caught in lethal traps were also necropsied in the laboratory following the same protocols. Wildlife species were identified to the genus level. Faecal samples were collected non-invasively from small carnivores, by keeping them in the trap for a maximum period of twelve hours. Non human primates were anaesthetised where trapped, using a combination of Medetomidine and Ketamine (under the supervision of a Kenya Wildlife Service veterinary officer), and morphometric data and a suite of biological samples (including faeces if available, or a rectal swab) were collected from each animal. The primate was carefully monitored throughout, and anaesthesia reversed using Atipamezol. Carnivores and NHPs were released unharmed at an appropriate time of day, from the same location at which they were trapped. Rodents were euthanized humanely for two reasons, (i) because they were trapped within people’s households and release of species that are deemed as pests (and a potential public health hazard) would not have been a viable option, (ii)In order to collect a fresh fecal samples via post-mortem.

Human and animal faecal samples were collected and transported on ice to one of two laboratories (University of Nairobi or Kenya Medical Research Institute) within five hours of collection. Questionnaires and data associated with samples was recorded using Open Data Kit (ODK) Collect software, on electronic tablets, and uploaded to databases held on servers at the International Livestock Research Institute (ILRI).

**Table S1.** Number and percentage (in brackets) of humans, individual wildlife and livestock sampled as part of the study.

| **Source** | **Frequency n (%)** |
| --- | --- |
| Human | 311 (23.63) |
| Livestock | 606 (46.05) |
| Bovine | 61 (4.64) |
| Chickens | 244 (18.54) |
| Duck | 25 (1.9) |
| Geese | 14 (1.06) |
| Goat | 109 (8.28) |
| Guinea fowl | 18 (1.37) |
| Pig | 49 (3.72) |
| Pigeons | 13 (0.99) |
| Sheep | 25 (1.9) |
| Turkey | 10 (0.76) |
| Rabbit | 38 (2.89) |
| Wildlife | 399 (30.32) |
| Avian | 245 (18.62) |
| Bat | 20 (1.52) |
| Primate | 4 (0.3) |
| Rodent | 130 (9.88) |

**Table S2.** Summary of AMR genes and point mutations classified by host type (human, livestock or wildlife). Numbers indicate the frequency and percentages (in brackets) of genes in host group (number of isolates with ARG present/total isolates). ARGs are ordered in descending order.

| **Antibiotic Resistance Genes** | **# Human (%, n=311)** | | **# Livestock (%, n=606)** | **# Wildlife (%, n=399)** |
| --- | --- | --- | --- | --- |
| sul2 | 168(54) | | 250(41.3) | 126(31.6) |
| strB | 154(49.5) | | 222(36.6) | 114(28.6) |
| strA | 155(49.8) | | 219(36.1) | 110(27.6) |
| tetA | 116(37.3) | | 239(39.4) | 113(28.3) |
| blaTEM_1B | 111(35.7) | | 122(20.1) | 69(17.3) |
| dfrA14 | 52(16.7) | | 119(19.6) | 58(14.5) |
| sul1 | 78(25.1) | | 77(12.7) | 44(11) |
| aadA1 | 36(11.6) | | 49(8.1) | 21(5.3) |
| dfrA7 | 44(14.1) | | 28(4.6) | 23(5.8) |
| dfrA1 | 32(10.3) | | 41(6.8) | 14(3.5) |
| tetB | 29(9.3) | | 35(5.8) | 20(5) |
| dfrA17 | 18(5.8) | | 29(4.8) | 22(5.5) |
| aadA5 | 18(5.8) | | 29(4.8) | 20(5) |
| gyrA_S83L | 26(8.4) | | 20(3.3) | 14(3.5) |
| catA1 | 28(9) | | 10(1.7) | 15(3.8) |
| qnrS1 | 13(4.2) | | 16(2.6) | 15(3.8) |
| dfrA5 | 12(3.9) | | 20(3.3) | 9(2.3) |
| aadA2 | 5(1.6) | | 18(3) | 13(3.3) |
| dfrA8 | 16(5.1) | | 7(1.2) | 4(1) |
| sul3 | 2(0.6) | | 14(2.3) | 16(4) |
| parC_S80I | 9(2.9) | | 8(1.3) | 9(2.3) |
| dfrA12 | 4(1.3) | | 17(2.8) | 8(2) |
| gyrA_D87N | 7(2.3) | | 6(1) | 7(1.8) |
| cmlA1 | 2(0.6) | | 8(1.3) | 9(2.3) |
| blaCTX_M_15 | 8(2.6) | | 3(0.5) | 4(1) |
| parC_A56T | 2(0.6) | | 13(2.1) | 4(1) |
| ant3_Ia | 3(1) | | 11(1.8) | 3(0.8) |
| aph3_Ia | 1(0.3) | | 7(1.2) | 6(1.5) |
| blaOXA_1 | 6(1.9) | | 3(0.5) |  |
| aac3_IId | 2(0.6) | | 2(0.3) | 4(1) |
| dfrA15 | 3(1) | |  | 2(0.5) |
| parE_I529L | 4(1.3) | | 1(0.2) |  |
| qepA4 | 2(0.6) | | 1(0.2) | 2(0.5) |
| parC_S57T | 2(0.6) | | 2(0.3) | 1(0.3) |
| parE_D475E | 2(0.6) | | 1(0.2) | 1(0.3) |
| fosA6 |  | | 1(0.2) | 3(0.8) |
| qnrB7 |  | | 1(0.2) | 3(0.8) |
| dfrA16 | 1(0.3) | |  | 2(0.5) |
| fosA3 |  | | 3(0.5) | 1(0.3) |
| qnrB2 |  | | 2(0.3) | 2(0.5) |
| gyrA_D87Y | 1(0.3) | |  | 2(0.5) |
| aac3_IIa | 2(0.6) | |  |  |
| blaTEM_30 | 2(0.6) | |  |  |
| blaTEM_33 | 1(0.3) | |  | 1(0.3) |
| blaTEM_70 | 1(0.3) | |  | 1(0.3) |
| catB3 | 2(0.6) | |  |  |
| qnrB19 | 2(0.6) | |  |  |
| aac6_Ib_cr | 2(0.6) | |  |  |
| gyrA_S83A | 2(0.6) | |  |  |
| parC_E84V | 2(0.6) | |  |  |
| blaCTX_M_14 | 1(0.3) | | 1(0.2) |  |
| floR |  | | 1(0.2) | 1(0.3) |
| parC_E84G | 1(0.3) | | 1(0.2) |  |
| aadA4 | 1(0.3) | |  |  |
| blaCMY_4 | 1(0.3) | |  |  |
| blaSHV_1 | 1(0.3) | |  |  |
| blaTEM_116 | |  |  | 1(0.3) |
| blaTEM_176 | |  | 2(0.3) |  |
| blaTEM_34 |  | |  | 1(0.3) |
| blaTEM_57 | 1(0.3) | |  |  |
| dfrA21 | 1(0.3) | |  |  |
| dfrB4 | 1(0.3) | |  |  |
| qnrB66 |  | |  | 1(0.3) |
| qnrS4 |  | |  | 1(0.3) |
| parC_E84A | 1(0.3) | |  |  |
| aph3_IIa |  | | 1(0.2) |  |
| aph6_Ic |  | | 1(0.2) |  |
| qepA1 |  | | 1(0.2) |  |
| parC_A108V |  | | 1(0.2) |  |

**Table S3.** Asymptotic estimates for the total number of AMR genes in humans, livestock and wildlife isolates and estimated sampling effort (function of observed richness and rarefied estimate)

| **Host** | Observed richness | Expected Richness | Estimated SE | 95% LCL | 95% UCL | Sampling effort (observed/ expected richness) (%) |
| --- | --- | --- | --- | --- | --- | --- |
| Human | 44 | 53.8 | 6.8 | 46.62 | 77.525 | 82 |
| Livestock | 38 | 54 | 14.837 | 41.725 | 112.5 | 70 |
| Wildlife | 39 | 47.09 | 7.092 | 40.843 | 74.535 | 83 |

**
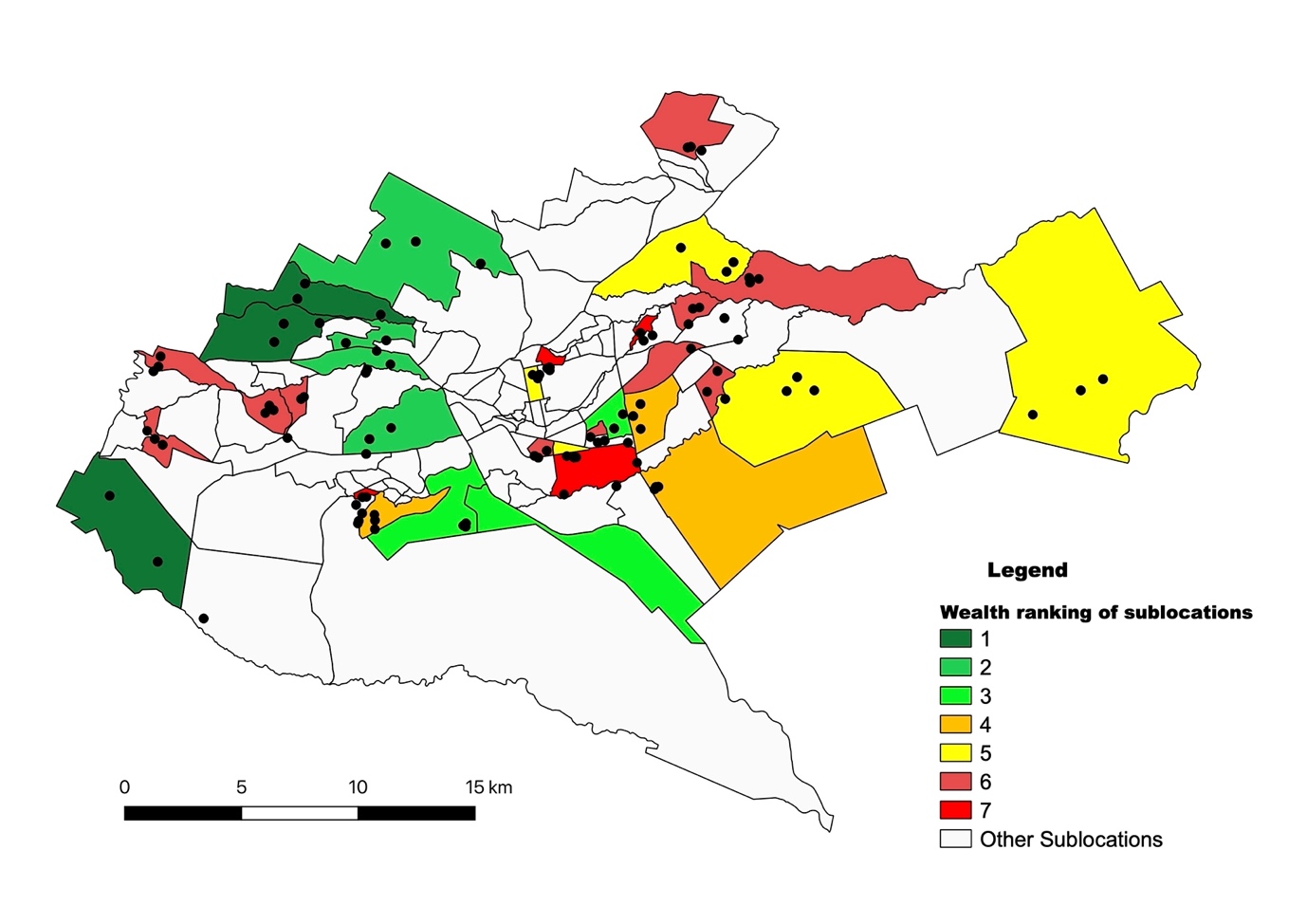
**

**Figure S1.** Geographical distribution of selected sublocations within the city of Nairobi chosen based on a socio-economic stratification, together with locations of each of 99 households selected within each stratum. Different colours given to the sublocations represent different wealth categories (Dark green – wealthy, dark red – poor)

**
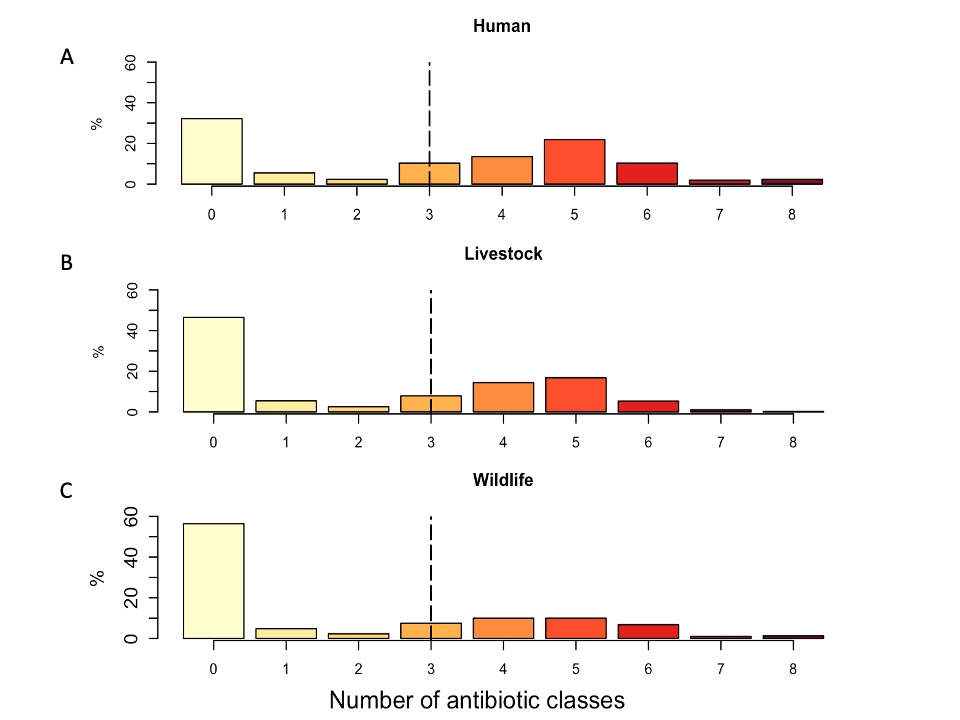
**

**Figure S2 (A-C)**. Histograms illustrating the number of antibiotic classes to which A) Human, B) Livestock, C) Wildlife *E. coli* isolates were detected as having AMR associated mechanisms. Dotted line shows the multi-drug resistance (defined as resistance to 3 or more antibiotic classes) threshold.

**
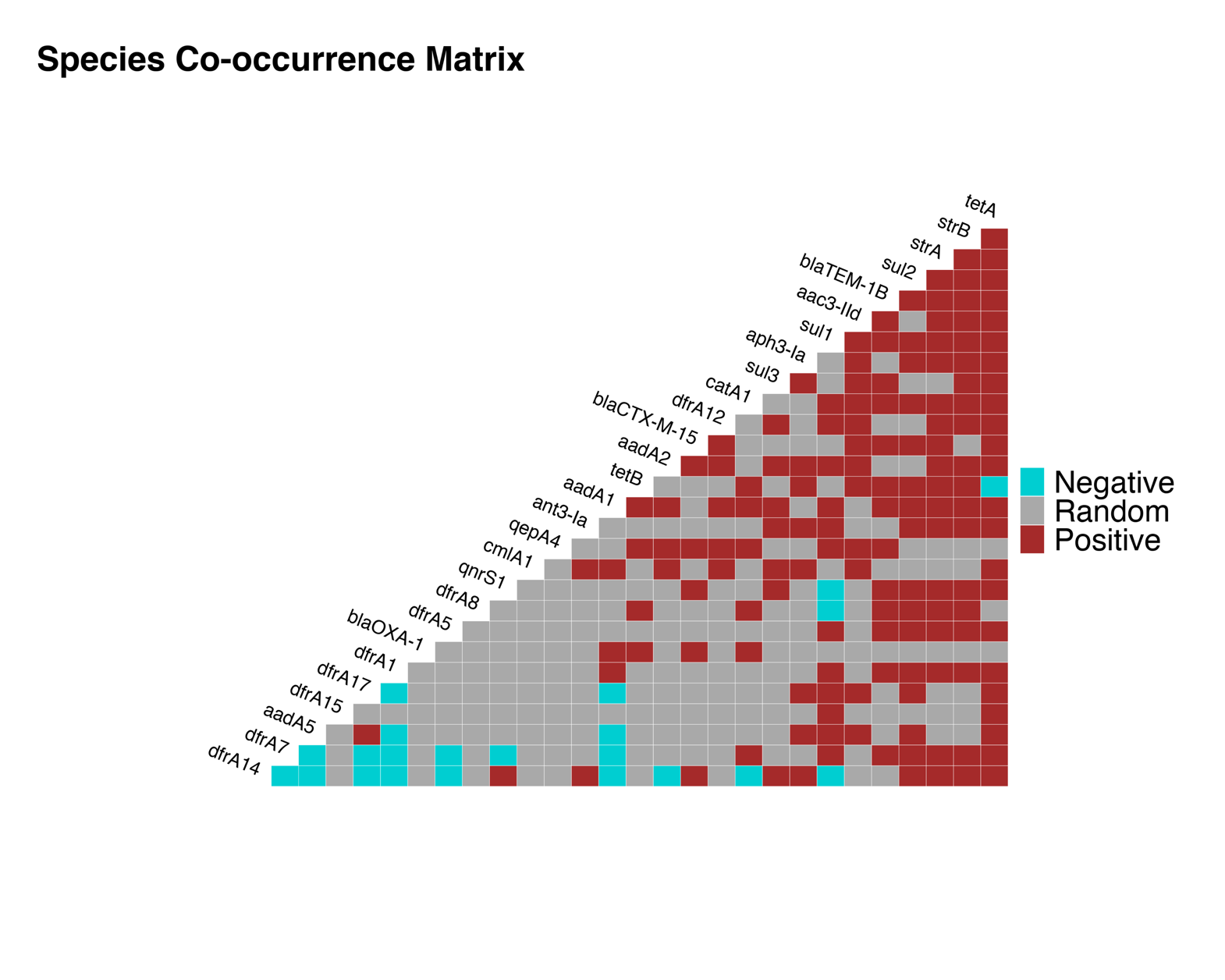
**

**Figure S3**. Heatmap of pairwise positive, negative and random associations calculated according to a probability co‐occurrence model for acquired AMR genes found in 0.5% of the isolates. AMR gene names are positioned to indicate the columns and rows that represent their pairwise relationships with other genes.

**Figure S4.** Network analysis revealing the AMR co-occurrence patterns among human, livestock and wildlife *E.coli* isolates. Lines join genes that co-occur together and colours represent the antibiotic class to egenes belong to (red - aminoglycosides, black - tetracylcines and yellow - betalactams).

**
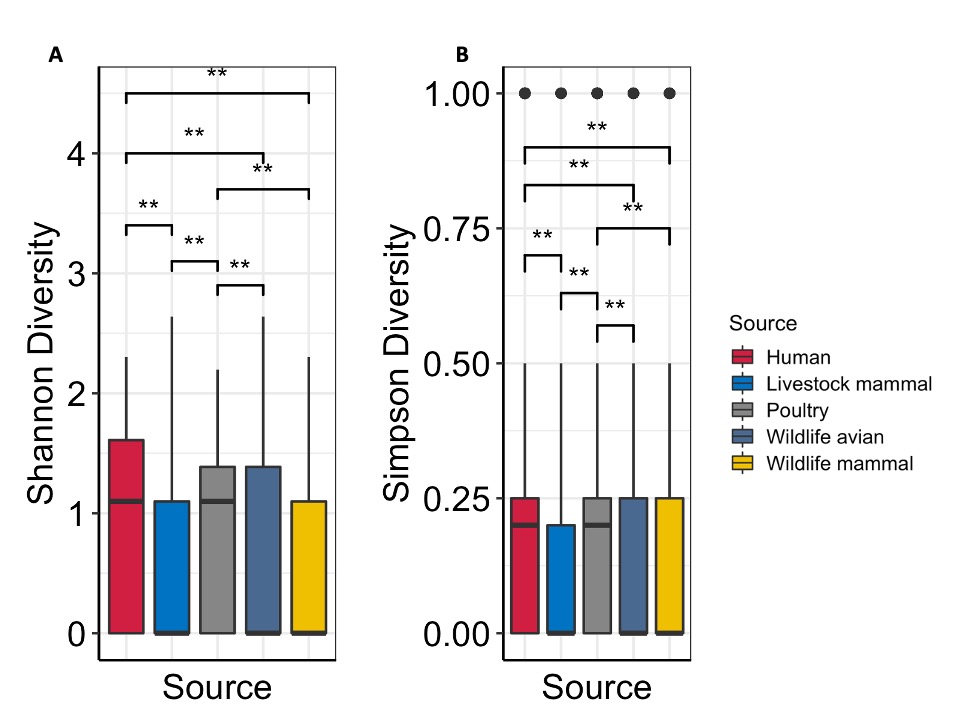
**

**Figure S5 (A-B).** Box-plots of A) Shannon diversity and B) Simpson index alpha diversity indices of acquired AMR genes. Data are shown via the interquartile ranges (IQRs) with the median as a black horizontal line and the whiskers extending up to the most extreme points within 1.5× the IQR; outliers are represented as dots. P values were calculated using the Kruskal Wallis test.
